# The complete mitochondrial genome of a wild-collected *Kappaphycus malesianus* (Solieriaceae, Rhodophyta)

**DOI:** 10.1080/23802359.2023.2183728

**Published:** 2023-03-10

**Authors:** Bea A. Crisostomo, Richard V. Dumilag, Michael Y. Roleda, Arturo O. Lluisma

**Affiliations:** a The Marine Science Institute, College of Science, University of the Philippines, Diliman, Quezon City, Philippines; b Fisheries Department, Sorsogon State University, Magallanes Campus, Aguada Norte, Magallanes, Sorsogon, Philippines; c Institute of Oceanography and Environmental Science, Mindanao State University, Tawi-Tawi College of Technology and Oceanography, Boheh Sallang, Sanga-Sanga, Bongao, Tawi-Tawi, Philippines

**Keywords:** Rhodophyta, mitochondrial genome, eucheumatoids, phylogenetics

## Abstract

*Kappaphycus malesianus* is a red seaweed farmed primarily for its carrageenan, a polysaccharide important in the food and pharmaceutical industries. Among the commercially cultivated *Kappaphycus* species, only *K. malesianus* has no mitogenome data available. Here, we assembled the mitochondrial genome of *K. malesianus* from next-generation sequencing data. The circular mitogenome consisted of 25,250 base pairs (bp) with a GC content of 30.25%. These values were comparable to previously sequenced solieriacean mitogenomes. Structural features, such as the stem-loop and hairpin, which were previously reported in other rhodophytes mitochondrial DNA, were also identified. The annotated genes (24 protein-coding genes, 24 tRNA genes, and 2 rRNA genes) were arranged in an order similar to the other available solieriacean mitogenomes. Lastly, phylogenetic analysis using 23 predicted protein domains showed the sister relationship of *K. malesianus* with other *Kappaphycus* species.

## Introduction

*Kappaphycus* and *Eucheuma* are the most commercially important eucheumatoids. These two carrageenan-producing rhodophyte genera belong to the family Solieriaceae. They are the primary source of commercial carrageenan, a phycocolloid used in various food, pharmaceutical and material products (Guo et al. 2022). The food-grade carrageenan market is seen to reach a value of USD 1.2 billion by the end of 2022, and is projected to increase to USD 2.3 billion in the next decade (Future Market Insights 2022). Euchematoids are morphologically plastic, and their cultivars are often recognized by farmers based on visible traits such as habit, color, and branching pattern (Dumilag et al. 2023). The study by Roleda et al. (2021) was unable to establish a link between genotype and morphology in *Kappaphycus* strains using the using *cox*1 and *cox*2-3 intergenic spacer sequences. Most cultivars were determined to belong to a single haplotype, which is likely due to the limited information provided by the short gene sequences (Lim et al. 2014; Dumilag et al. 2023). With the availability of genomic data, more information would be available, leading to the possibility of developing SNP markers beyond what is currently used. Among the commercially cultivated eucheumatoids, *Kappaphycus malesianus* J. Tan, P.E. Lim & S.M. Phang 2013 has been the least studied and has no genome data available. In this study, we present the assembled and annotated mitochondrial genome of *K. malesianus*.

## Materials and methods

**Figure 1.**
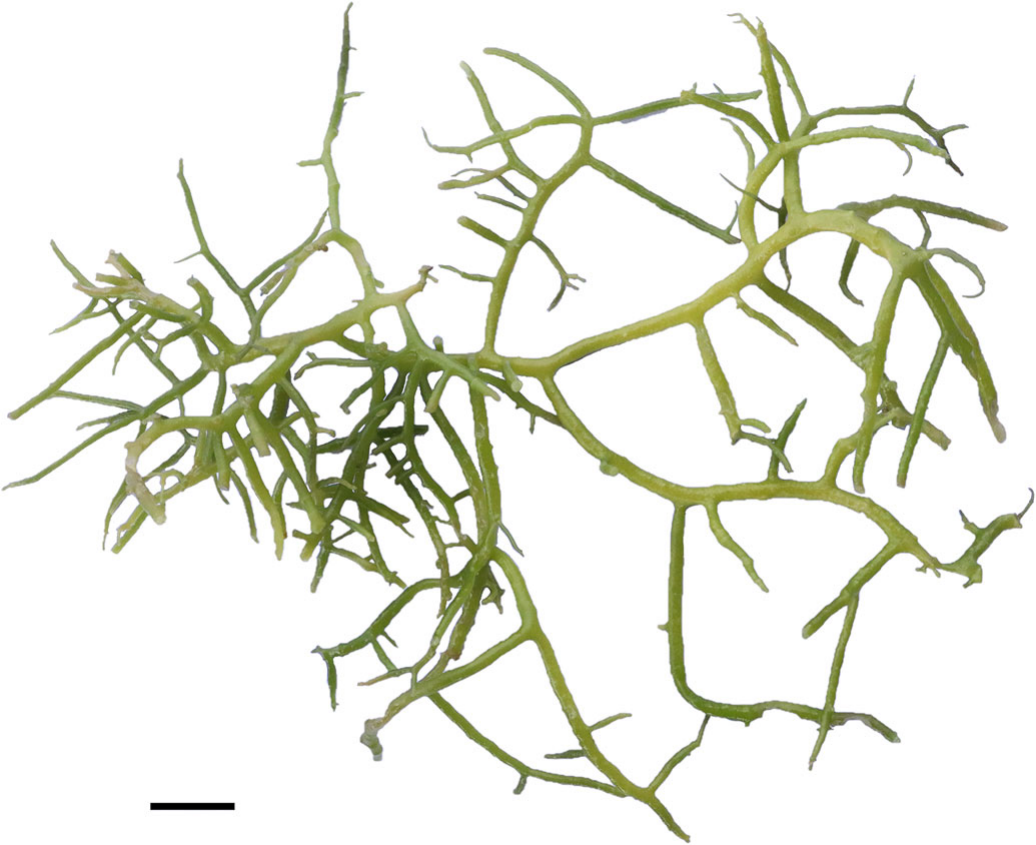
The habit of the *K. malesianus* specimen RD1484 (scale bar = 5.0 cm). Photo credits: R. V. Dumilag.

With the approval and supervision of the Local Government Unit (LGU) of Sitangkai, a specimen of *K. malesianus* was collected from Sapa-Sapa Bank, Sitangkai, Tawi-Tawi, Philippines (4°43'11.6’N 119°12'20.0’E). The voucher specimen (Figure 1) was identified as *K. malesianus* by R.V. Dumilag then deposited in the Mindanao State University Herbarium (MSU; msuh@msutawi-tawi.edu.ph; http://sweetgum.nybg.org/science/ih/herbarium-details/?irn=259064) with the voucher number RD1484. DNA was extracted using a combination of modified CTAB (Zuccarello et al. 2006) and MagAttract HMW DNA kit (Qiagen) following the manufacturer’s protocols. The extracted DNA sample was sent to BGI Hong Kong for Library preparation using Illumina DNA PCR-Free Prep (Illumina, USA) and sequencing (150PE) using the Novaseq6000 platform (Illumina, USA). The complete mitogenomes of *Kappaphycus striatus* (NC024265) and *Kappaphycus alvarezii* (NC031814) were used as references during assembly and annotation. The genome was assembled using GetOrganelle (Jin et al. 2020) and the coverage depth was visualized using Bandage v0.8.1 (Wick et al. 2015). Annotations were done using AGORA (Jung et al. 2018) and tRNAscan-SE (Lowe and Chan 2016). Secondary structures were predicted using the RNAfold web server (Gruber et al. 2008). The final genome map was generated using OGDRAW (Greiner et al. 2019). To compare the gene order, Mauve 2.4.0 (Darling et al. 2004) was run using the progressive mode. For phylogenetic analysis, 23 mitochondrial protein sequences predicted from genes (*atp*4, *atp*6, *atp*8, *atp*9, *cob*, *cox*1, *cox*2, *cox*3, *nad*1, *nad*2, *nad*3, *nad*4, *nad*4L, *nad*5, *nad*6, *rpl*16, *rps*3, *rps*11, *rps*12, *sdh*2, *sdh*3, *sdh*4, and *tat*C) were aligned using Clustal Omega (Madeira et al. 2022) then concatenated. Gblocks 0.91b (Castresana 2000; Dereeper et al. 2008) was used to remove highly divergent regions of the alignment. The best-fit substitution model was selected using the IQ-TREE webserver (Trifinopoulos et al. 2016) and was determined to be MtMet + F + I + G (Le et al. 2017) using the Akaike information criterion (AIC). Phylogenetic trees were then inferred using maximum likelihood (ML) as implemented by IQ-TREE, and Bayesian inference (BI) using MrBayes 3.2.7 (Ronquist et al. 2012). The model used for BI was CpRev + I + F + G (Adachi et al. 2000) since MtMet was not available in MrBayes. In addition to the eucheumatoid sequences (Tablizo and Lluisma 2014; Li et al. 2018), sequences from the closely related species, *Mastocarpus papillatus* (Sissini et al. 2016), *Riquetophycus* sp. (Yang et al. 2015), and *Chondrus crispus* (Leblanc et al. 1995), were included in the phylogenetic tree construction.

## Results

**Figure 2.**
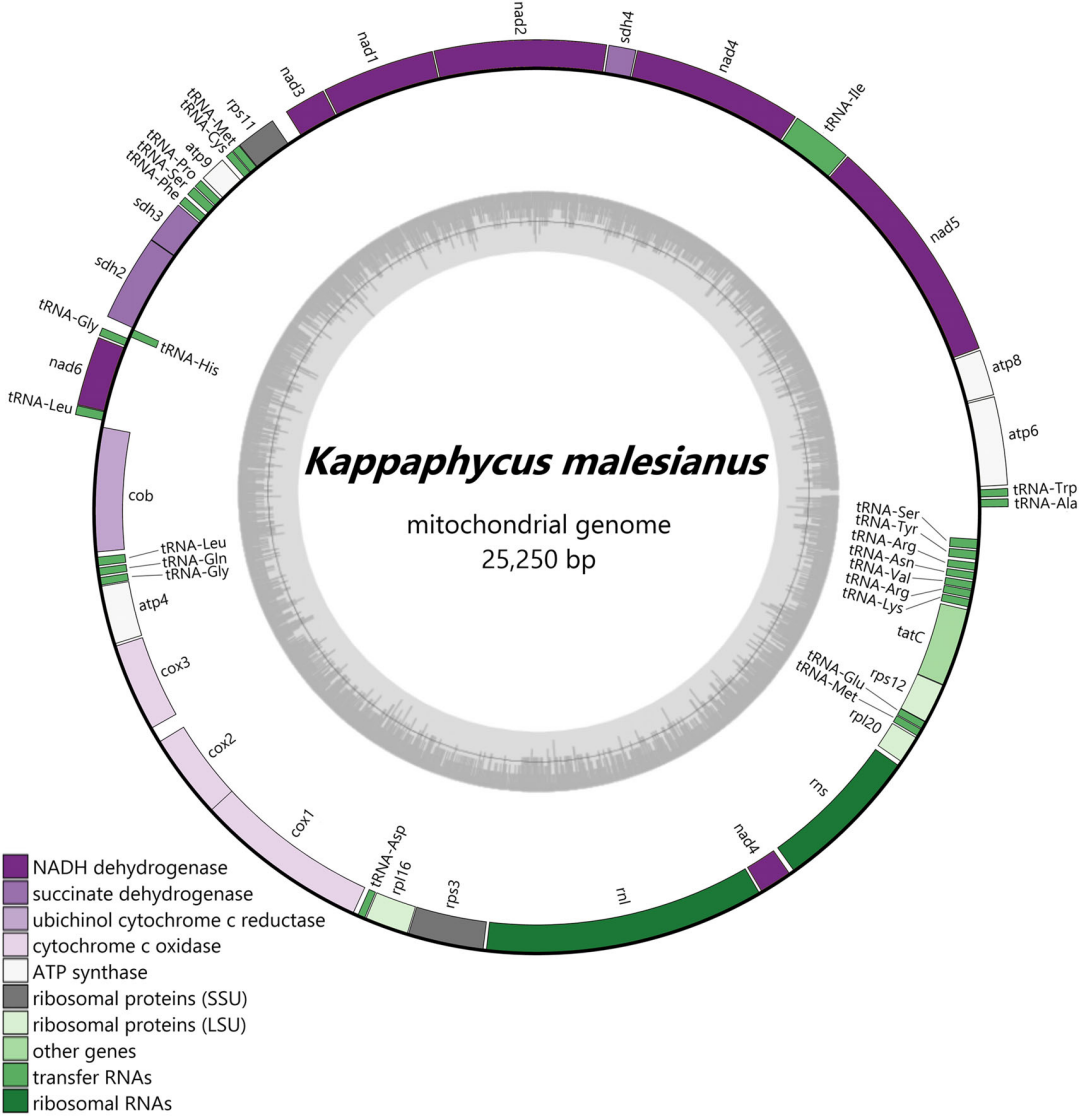
Map of *K. malesianus* mitochondrial genome. Annotations outside the circle are in forward orientation, while those inside the circle are in reverse orientation. The GC content graph is illustrated inside the circle in gray.

The *K. malesianus* mitogenome was assembled into a single circular structure of 25,250 bp with a depth of 144.7x (Figure S1) and a GC content of 30.25% (Figure 2). Its size was longer than that of *K. alvarezii* and *K. striatus* (Tablizo and Lluisma 2014; Li et al. 2018) by 52 and 8 bp, respectively. The total number of genes was 50 (24 protein-coding genes, 24 tRNA genes and 2 rRNA genes), representing ∼94% of its total length (Tables 1, S1 and S2). These genes were distributed almost evenly between the two mitochondrial DNA (mtDNA) strands, forming two transcriptional units in opposing directions (Figure 2). At the junction of these units were two AT rich inverted repeats, which were also present in all the sequenced rhodophyte mitogenomes (e.g. Li et al. 2018). In the *K. malesianus* mitogenome, a 98-nucleotide stem-loop (ΔG = −67.70 kcal/mol) was found between the *trn*S2 and the *trn*A genes, while a 47-nucleotide hairpin (ΔG = −34.73 kcal/mol) was found between the *cob* and the *trn*L1 genes. The predicted protein products and their order appeared to be conserved within congeneric species (Figure S2). Similar to the other eucheumatoids, only 19 types of tRNAs were identified since the tRNA for threonine (Thr) was missing (Tablizo and Lluisma 2014; Li et al. 2018). The features of *K. malesianus* mitogenome as compared with those of other eucheumatoids are presented in Table 1.

**Table 1.** General features of the mitochondrial genomes of five eucheumatoid species.

	*B. gelatinus* ^a^	*E. denticulatum* ^a^	*K. alvarezii* ^a^	*K. malesianus* ^b^	*K. striatus* ^c^
Genome size (bp)	25,275	25,327	25,198	25,250	25,242
GC content (%)	29.7	30.3	29.86	30.25	30.06
Protein-coding (%)	70.23	70.15	70.29	70.17	70.21
Spacer content (%)	4.85	5	4.96	5.54	5.13
Total number of genes	50	50	50	50	50
Protein-coding genes	24	24	24	24	24
tRNA genes	24	24	24	24	24
rRNA genes	2	2	2	2	2
Intron	1	1	1	1	1

^a^Li et al. (2018)

^b^This study

^c^Tablizo and Lluisma (2014)

**Figure 3.**
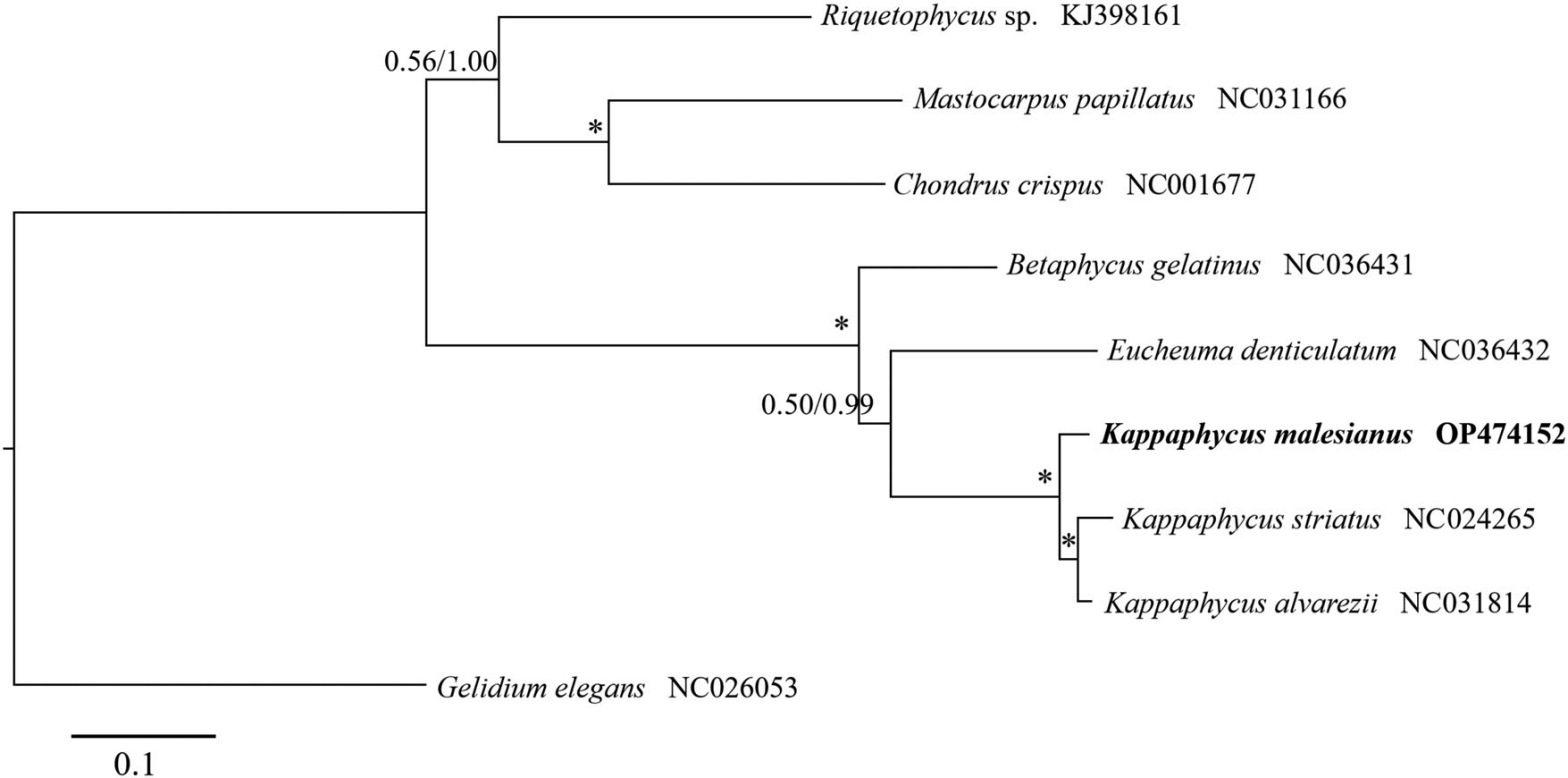
Maximum likelihood tree of nine rhodophytes based on 23 predicted mitochondrial proteins. The numbers above each node represent support values calculated from 1000 maximum likelihood bootstraps (left) and Bayesian posterior probability (right). Asterisk indicates a value of 1.00 for both supports. The scale bar indicates the number of substitutions per site.

Phylogenetic analysis shows that *K. malesianus* is related to *K. alvarezii* and *K. striatus* (Figure 3). Similar to previous reports, which used barcoding markers (Lim et al. 2014), our findings indicated stronger support of the sister relationship of *K. alvarezii* and *K. striatus* than that of *K. malesianus* with either taxon.

## Discussion and conclusion

The structure and organization of all the available *Kappaphycus* mitogenomes were conserved. The phylogenetic relationships of the farmed eucheumatoids inferred from mitochondrial proteins were consistent with previous DNA barcoding studies. Currently, the Indo-Pacific eucheumatoids are divided into five genera: *Eucheuma*, *Betaphycus*, *Mimica*, *Kappaphycopsis*, and *Kappaphycus* (Dumilag and Zuccarello 2022), with still unresolved intergeneric relationships. The inclusion of mitogenomes of other eucheumatoid taxa in future phylogenetic studies may resolve intergeneric relationships within the eucheumatoids.

## Supplementary Material

Supplemental Material

## Data Availability

The mitogenome sequence data that supported the findings in this study are openly available in GenBank of NCBI at (https://www.ncbi.nlm.nih.gov/) under accession no. OP474152. The associated BioProject, SRA, and Bio-Sample numbers are PRJNA899665, SRR22244159, and SAMN31665931, respectively.
