# Supplementary figures and images for: The complete mitochondrial genome of a wild-collected Kappaphycus malesianus (Solieriaceae, Rhodophyta)
Source: Mitochondrial DNA B Resour. 2023 Mar 10;8(3):359–63. doi: 10.1080/23802359.2023.2183728 (PMC10013388; doi:10.1080/23802359.2023.2183728)

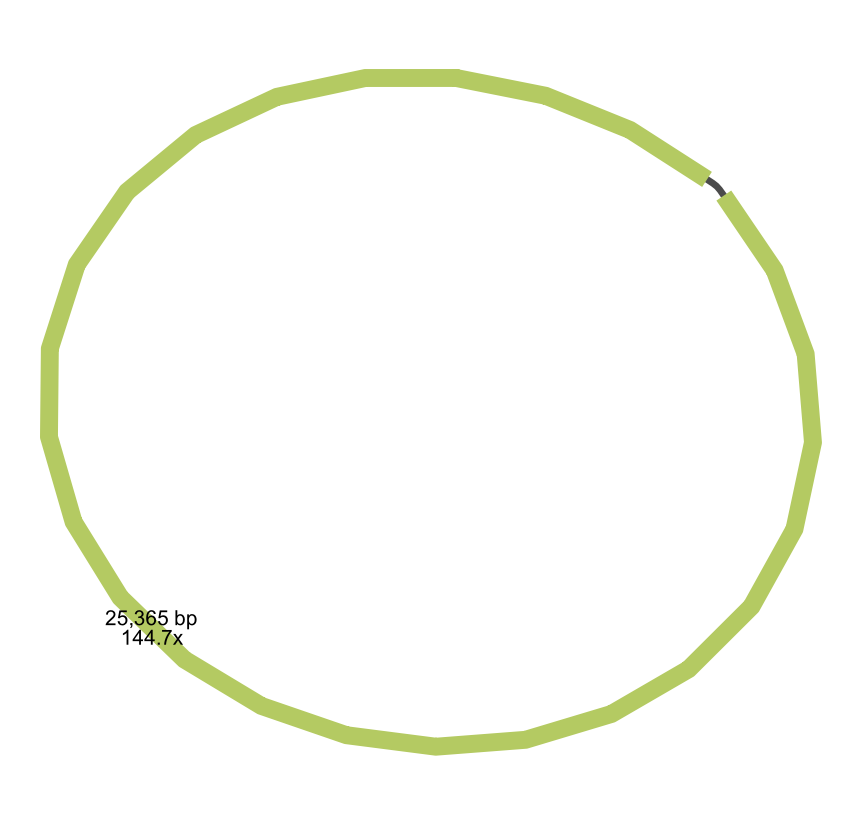

Supplement: Supplemental Material [file TMDN_A_2183728_SM1235.zip › suppl_data/Kmalesianus_mitogenome_FigS1.png]

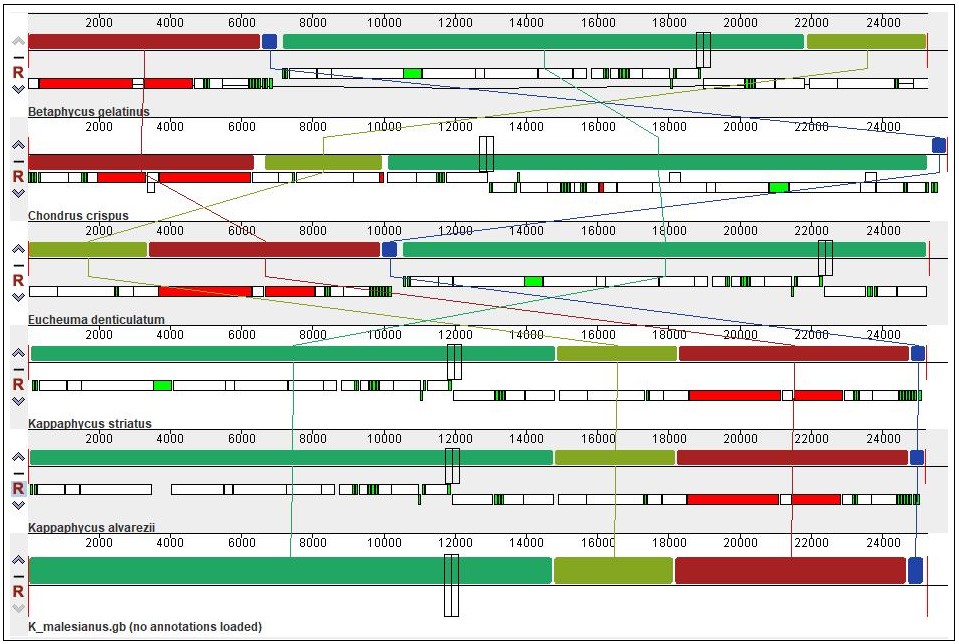

Supplement: Supplemental Material [file TMDN_A_2183728_SM1235.zip › suppl_data/Kmalesianus_mitogenome_FigS2.jpg]
